# Systemic Problems: A perspective on stem cell aging and rejuvenation

**DOI:** 10.18632/aging.100819

**Published:** 2015-10-25

**Authors:** Irina M. Conboy, Michael J. Conboy, Justin Rebo

**Affiliations:** ^1^ Department of Bioengineering, UC Berkeley; QB3, CA 94709, USA

**Keywords:** parabiosis, aging, rejuvenation, stem cells, neurogenesis

## Abstract

This review provides balanced analysis of the advances in systemic regulation of young and old tissue stem cells and suggests strategies for accelerating development of therapies to broadly combat age-related tissue degenerative pathologies. Many highlighted recent reports on systemic tissue rejuvenation combine parabiosis with a “silver bullet” putatively responsible for the positive effects. Attempts to unify these papers reflect the excitement about this experimental approach and add value in reproducing previous work. At the same time, defined molecular approaches, which are “beyond parabiosis” for the rejuvenation of multiple old organs represent progress toward attenuating or even reversing human tissue aging.

## Prologue

How long has it been since we knew that age-imposed changes in the circulatory milieu are to blame for the progressive attrition of organs and degenerative disorders that invariably accompany human aging? Some say, we've known for millennia, from the Ancient Greeks and Medieval stories of vampires. Others say that it was McKay's 1950s experiments, where old rats were sutured with young rats to establish parabiosis, aka, a joined blood circulation that suggested better health of largely non-cellular cartilage [2].

**Figure 1:**
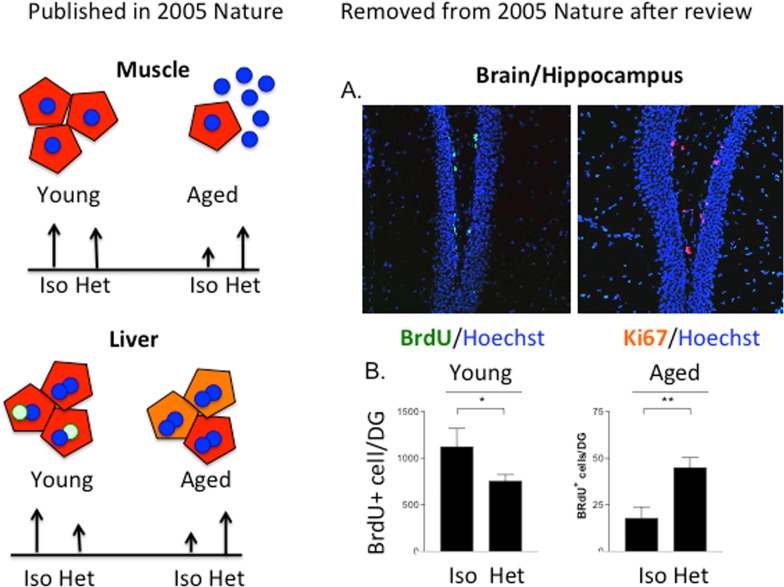
The age of the systemic milieu affects myogenesis, hepatogenesis AND neurogenesis The work on myogenesis and liver regeneration is described in [1]. The work on neurogenesis was reviewed as a part of the same manuscript (conceived, planned, executed and written by Irina and Michael Conboy and Tom Rando; Conboy et al, 2004-02-15708A) but was removed after the review. (**A**) BrdU was administered to 5 week parabionts at 5, 3 and 1 day before mice were sacrificed and brains were processed for immunofluorescence analysis. Sections through the dentate gyrus of the hippocampus were immunostained for BrdU (green; left panel) or Ki67 (red; right panel) to identify proliferating progenitor cells. Hoechst dye (blue) labels all nuclei. (**B**) Quantitation of neural stem cell proliferation as determined from experiments represented in panel (**A**). Counting BrdU+ cells in periodic sections and extrapolating to the entire thickness of the DG estimated the total number of proliferating neural stem cells. The scales for the young and aged animals are different because of the marked global reduction in neurogenesis in brains of old mice compared with young mice, as previously reported. Neural stem cell proliferation was significantly enhanced for old partners in heterochronic pairings, compared to those in isochronic pairs (**p < 0.005). There was an inhibition of proliferation in young partners involved in heterochronic pairings compared to those in isochronic pairings (*p < 0.05).

Yet, another answer is that it has been 10 years since the paradigm-shifting observations that in heterochronic parabiosis, the young systemic milieu rapidly and broadly rejuvenates organ stem cells in muscle, brain/hippocampus and liver, while the old systemic milieu rapidly and broadly ages myogenesis, liver regeneration and neurogenesis, with the responsible biochemical pathways being re-set to their young or old states ([1], and Figure 1).

## What paradigms have been shifted?

Before this work, the prevalent theories of tissue decline in aging focused on cumulative cell intrinsic changes as culprits: telomere attrition, DNA damage, oxidative damage, mitochondrial dysfunction, etc.). While all of the above continue to be true for differentiated cells, it is important to realize that organ stem cells age “extrinsically” [1, 4–7], and maintain a relative “youth” that could be due to the state of quiescence, which is default for most if not all postnatal stem cells [10]. As such, stem cell regenerative capacity persists throughout life, but sadly, the biochemical cues regulating organ stem cells change with age in ways that preclude productive regenerative responses, causing the abandonment of tissue maintenance and repair in the old [3].

Promisingly, numerous studies have demonstrated that experimental youthful re-calibration of specific biochemical cues will quickly (within days) rescue the effective regenerative capacity of old stem cells in *vivo*, demonstrating that old stem cells can for all practical purposes maintain old organs [4, 11–14]. Such quick and robust “rejuvenation” also suggests that not much intrinsic “aging” has been experienced by these stem cells, or that the intrinsic aging of stem cells can be rapidly reversed (within 24 hours) after exposure to youthful molecular cues, for example, by activation of Notch [4]. Conversely, progeric changes in these defined bio-chemical signals make even young stem cells behave like old in a day, before the byproducts of metabolic stress or environmental damage or prolonged entropy could accumulate [3, 5, 7, 14].

In support of the notion of a maintained, intrinsic “youth”, old muscle stem cells do not accumulate DNA damage with age [15] and preserve youthful telomerase activity [16]. Hence, any new muscle tissue that is produced is likely to be healthy. Notably, a genetic deficiency in DNA repair doesn't seem to affect muscle regeneration. Indeed, young muscle stem cells from SCID mice, which are deficient in non-homologous end joining repair, do accumulate more DNA damage than wild-type but their muscle regeneration is as effective as young wild-type, evidence further uncoupling DNA damage from stem cell aging [15].

## If all this has been known for 10 years, why is there still no therapeutics?

One reason is that instead of reporting broad rejuvenation of aging in three germ layer derivatives, muscle, liver, and brain by the systemic milieu, the impact of the study published in 2005 became narrower. The review and editorial process forced the removal of the neurogenesis data from the original manuscript. Originally, some neurogenesis data were included in the manuscript but, while the findings were solid, it would require months to years to address the reviewer's comments, and the brain data were removed from the 2005 paper as an editorial compromise. The phenomenon and its magnitude were replicated, expanded and elegantly described in a 2011 paper [17]. If the friendly neighbor of the Rando lab had not been interested in this project after learning of our findings, this important result could have been lost or remained on a “back-burner” indefinitely. While it is certainly better late than never and even if the data are more elaborated than in the original manuscript, one can argue that the scientific community could already have been working on the extrapolation of these results and translating them into therapeutics against neuro-degeneration for 10 years.

Another reason for the slow pace in developing therapies to broadly combat age-related tissue degenerative pathologies is that defined strategies, which are “beyond parabiosis”, for the rejuvenation of multiple old organs have been very difficult to publish in high impact journals; only the magic of “heterochronic parabiosis” seems to keep the editors' and reviewers' attention. As the result, in the current dynamically raging scientific waters, significant work that is directly relevant to attenuation or even reversal of human tissue aging (e.g., molecules that work in mice and are FDA approved or in clinical trials for human applications) can sadly get washed over, particularly, when relevant publications from lower impact journals are not always noticed or cited. At the same time, many highlighted recent reports on systemic tissue rejuvenation combine parabiosis with a “silver bullet” putatively responsible for the positive effects. Attempts to unify these papers (e.g. [18]) reflect the excitement about this experimental approach.

## What about other potential influences of heterochronic parabiosis?

The old parabiont benefits from not just young blood, but also the young organs: heart, lungs, liver, kidneys, thymus, etc; and removal/neutralization by the young parabiont of negative metabolites, chemokines, etc. These together with improved blood oxygenation, normalized glucose/insulin and cholesterol profile are all likely to contribute to the rejuvenated tissue stem cells [3, 19]. The young parabiont has to maintain an additional aged body with poorly functioning organs, inflammation, ongoing pathologies and perturbed immune responses, which could all contribute to the observed slight decline of the young stem cell responses.

The old parabiont has a much more stimulating environment when sutured with a young rather than an old partner. In contrast to the old and more sedentary animals, young mice are active and the old partner literally has to tag along. The pheromone landscape also becomes changed in hetero-, as compared to isochronic pairs. It is known that pheromones as well as environmental enrichment enhances, whereas environmental deprivation decreases neurogenesis and neuronal plasticity [20, 21] and “mock parabioses” have not been done to control for this.

Blood cells in parabiotically shared circulation do not trans-differentiate to make muscle, brain, etc., tissues [22–25]; however, their cytokines certainly influence organ stem cell performance [26–28]. Additionally, wound clearance by the leukocytes (that becomes inefficient with age) is quite important for successful regeneration of injured tissues, and tissues of the old parabiont would benefit from the young leukocytes [29].

Young and old blood serum and its chemical components have inducing/inhibitory properties *in vitro* on stem cells freshly isolated from mice and humans [1, 30]. Molecules induced by injury to solid tissues “prime” remote organ stem cells in an endocrine fashion [31], while stem cell inhibitory proteins such as osteopontin become elevated in circulation after tissue injury in old mice [12]. Thus, some serum-related effects are expected to play a key role in stem cell aging and rejuvenation. However, these need to be experimentally uncoupled from all the other above-mentioned influences in parabiotically joined animals.

## Is it wise to search mostly for young plasma fractions and factors?

One conclusion from the heterochronic parabiosis studies is that the regenerative capacity of old tissue stem cells can be enhanced by the young systemic milieu; however, an over simplistic vision that using small volumes of young plasma or a “systemic silver bullet” will provide rejuvenation, e.g. one circulating molecule, at this point seems unlikely. Aging is a multi-genic process, the list of potential “silver bullets” is short, and some are oncogenic. Notably, while administration of small volume of young plasma to aged mice improved their cognition, the effects on brain or other tissue *stem cells* or health span have not been studied [32]. Most importantly, the positive effects of young blood on old are only partial for muscle and the increase neurogenesis is nowhere near levels seen in young brain (Figure 1, [1, 17, 32, 33]). Moreover the strong *inhibition of young tissue stem cells by the aged systemic milieu in vivo* and by old serum *in vitro* have been repeatedly reported [3, 34–36]. Summing up what is known, introducing small volumes of young plasma into an old host may not work effectively for enhancing tissue regeneration in the old, unless the inhibitory components of the aged circulation are neutralized or removed. And notably, removal or neutralization of these inhibitory systemic factors is predicted to have a positive effect on tissue repair by itself [6, 11, 19, 37].

**Figure 2:**
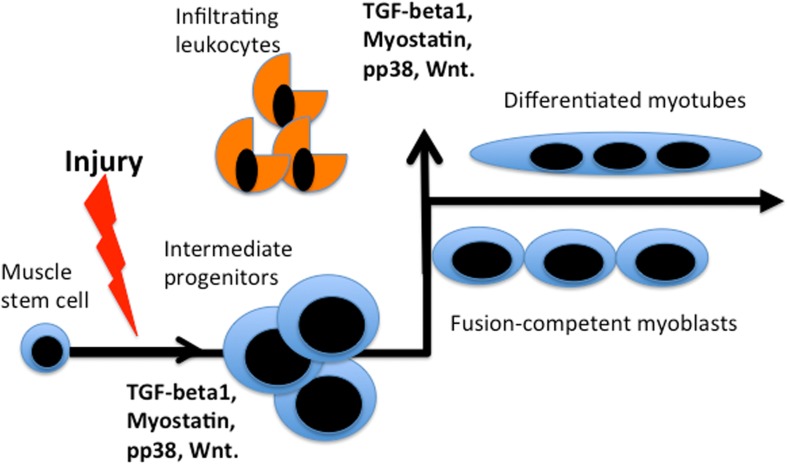
Bi-phasic requirements for TGFβ, myostatin, pp38, Wnt during muscle repair Activated by muscle injury quiescent muscle stem cells enter cell cycle and differentiate into rapidly dividing intermediate progenitor cells that expand, when the levels of listed factors are low. Up-regulation of these factors (likely by many sources, including infiltrating leukocytes that clear the wound) is needed for productive differentiation of the intermediate progenitors into fusion-competent myoblasts and post-mitotic multi-nucleated myotubes. *In vitro*, intermediate progenitors can be expanded in high mitogen medium and upon withdrawal of mitogens they form multi-nucleated myotubes in culture.

**Figure 3:**
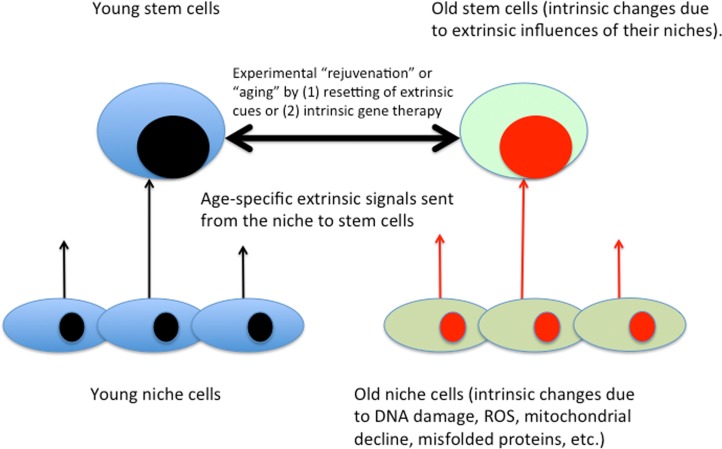
Intrinsic / extrinsic aging of tissue stem cells Based on the body of published literature, gene expression, epigenome, signal transduction and behavior of stem cells are influenced by the signals that emanate from their local and systemic niches. Niche cells experience intrinsic aging, which results in the changed extrinsic influences on tissue stem cells. Accordingly, in addition to gene therapy and not adding stem cells, defined molecular approaches for enhancement of tissue maintenance and repair have been demonstrated [3].

Additionally, it is not clear which *levels* of the circulatory molecule(s) are necessary and sufficient for the pro-regenerative activity of the old stem cells. Many factors, such as TGF-beta1, Wnt, or pp38α become inhibitory at the excessive levels seen with age but are needed for normal cell and tissue homeostasis at their young levels and furthermore, are biphasic during the regenerative process (Figure 2 and [3, 30, 37–39]).

## GDF11 abdicates the throne of systemic rejuvenation

As recently revealed, the key findings on diminished levels of GDF11 with age and of the positive effects of this protein on myogenesis are not reproduced [36, 40–42]. Very importantly, this would not come as a shock if one would read the relevant papers. Based on the science, GDF11 is 90% identical to myostatin, which inhibits (not activates) myogenesis. GDF11 in neurogenesis serves to limit the numbers of neural stem cells [43, 44] and this discrepancy was not cited or discussed in the pro-rejuvenation papers. GDF11 signals through the same receptor as TGF-beta1 and myostatin (more efficiently than myostatin [40] and inhibition (not activation) of this ALK5 receptor has been shown to enhance and rejuvenate myogenesis and also, neurogenesis [6, 37]. The bulk of data questions the anti-aging effects of GDF11, and makes the *Cell Metabolism* paper important for avoiding years of unproductive research in the wrong directions.

GDF11 was reported to enhance old muscle repair by attenuating the accumulation of DNA damage in the aged satellite cells [36]; however, a year earlier it was published that old muscle satellite cells do not accumulate DNA damage with age and that DNA damage in muscle stem cells is uncoupled from efficiency of muscle repair and likely represents a physiologically required process of terminal myogenic lineage differentiation [15], [45]. Once again, a key paper in a lesser impact journal questioning main conclusions of a high impact paper was not cited or caught in peer or editorial review.

In apparent disagreement between two recent studies, GDF11 does [41] and does not [46] reduce hypertrophy of old mouse hearts. Even if there is no direct connection to aging, stem cells or heterochronic parabiosis, ectopic GDF11 may reduce the mass of both young and old hearts [47].

Age-specific levels of GDF11 are also not without controversy: initially, it was reported that GDF11, but not GDF8/myostatin, declines with age [41]; then more recently it was determined that GDF8/myostatin does decline with age [48], an observation which was also reconfirmed by the original *Cell* authors, who now combine the two protein names into one, called GFD11/8 [47].

These controversies may be caused by the fact that antibodies to GDF11 have cross-reactivity with GDF8/myostatin. GDF11 antibody might also simply cross-react with immunoglobulins, which become elevated with age [47, 49]. A very elegant recent paper using a clean myostatin knock-out system demonstrated that GDF11 levels are 500 times less than that of myostatin or activin, thus precluding any competitiveness for the same receptor complexes and arguing against physiological modulation of pSmad2/3 signaling by GDF11 when myostatin, activin or TGF-beta1 are present [48].

Muscle tissue produces myostatin, so the age-specific decline in myostatin protein may reflect the loss of muscle mass in the old. Consequentially people or mice with physiologically higher muscle mass (particularly in older age) are likely to be healthier, thus exhibiting an indirect link between the levels of myostatin and the health of the heart and other organs. Notably, GDF11 is associated with human colon cancers, which is likely due to the pro-angiogenic properties of GDF11 and most TGF-beta family ligands [50, 51]. Accordingly, Alk1 and Alk5 inhibitors are studied as anti-cancer anti-angiogenic blockers [52, 53].

## Extrinsic versus intrinsic is semantics

If p38 becomes up regulated or telomere maintenance inhibited in cells with age, because TGF-beta in their environment becomes elevated, should this be considered intrinsic or extrinsic aging [54]? Accordingly, if either inhibition of p38 or of TGF-beta signaling improves myogenicity, was the intrinsic or extrinsic aging reversed? A number of recent papers [14, 55–57] reveal persistent age-specific changes in muscle stem cells, but the data shown in these manuscripts does not directly dispute the original findings that in hetero-chronic muscle transplants both young and old cells do worse in the old host than in young [58]. Moreover, in the intrinsic-focused papers the regenerative capacity of the aged stem cells is quickly enhanced, for example, by changes in phosphorylation of intracellular proteins meaning that such “aging” is acutely reversible, in agreement with heterochronic parabioses. Even with respect to the induction of p16 and of muscle stem cell senescence with advancing age, experimental recovery into more youthful phenotypes have been reported, albeit it remains to be seen if there is a dilution of irreversibly senescent cells by the boost of pre-senescent cell proliferation, or a bone fide reversion of the senescence program in cells [57, 59, 60].

Namely, inhibitors of p38, a determinant that restricts proliferation and promotes differentiation of muscle progenitors [61] enhanced the transplantation efficiency of old myogenic cells [55, 59]. p38 α, β, γ and δ isoforms are differentially expressed in various cells [61, 62] and interestingly, unlike the isoform α, p38 β or γ KO did not alter adult myogenesis [63]. Significant recent work established conservation in the regulatory myogenic activity of p38 between mouse and human muscle stem cells [64]. p38 levels are perturbed in the old and pathological cells due to a number of extrinsic and intrinsic cues, including the ROS-related DNA damage and perturbed mitochondrial activity; and p38 inhibitors have been hence, examined for many years [65–69].

It is well known that the intracellular changes (be those in phosphorylation status of proteins, gene expression or epigenome) are regulated by the signals emanating from stem cell niches and that such signals change with age in ways that inhibit engagement of stem cells in tissue maintenance and repair. The regulation of stem cell homeostasis by differentiated niches is evolutionary conserved between such distinct species as flies, mice and humans [3, 70–72]. The semantics of intrinsic (autonomous) versus extrinsic aging of stem cells is illustrated in Figure 3.

Even for the stronghold of “autonomous aging”, hematopoietic stem cells (HSCs) [73, 74], a combination of both intrinsic and extrinsic age-related changes seems to be in place [75, 76]. In addition to wet lab studies, the age-imposed extrinsic influences on HCSs have been modeled mathematically [77, 78]. The relative contribution of niche-directed versus intrinsic aging may, of course, differ in different regenerative cells: for example, the levels of aberrantly processed Lamin A (progerin) increase with age in bone marrow adult multilineage cells (also known as mesenchymal stem cells) where experimental induction of progerin in young cells, significantly disrupts the expression and localization of self-renewal markers and the regenerative capacity of cells, in part through deregulation of Oct1, and subsequent perturbation of the mTor pathway and autophagy [79, 80].

On a related note, while a consensus has not yet been reached on whether and to what degree muscle stem cells are lost with age, and if these cells are exhausted via proliferation or in contrast, up regulate CDK inhibitors, keep their loci epigenetically open and fail to proliferate [15, 55, 60, 81, 82], there is general agreement that regeneration and maintenance of multiple organs can be enhanced in old mammals by boosting performance of their own aged stem cells, without ectopic gene expression or cell transplantation [3, 10, 14, 42].

In the same vein, mature myofibers are known to synthesize protein and increase in size by hypertrophy [83], which still occurs when 85–90% of satellite cells are ablated [81]. Despite semantics, this does not exclude contribution of muscle stem cells to this tissue even in sedentary mice [84] or the effects of their functional decline with aging particularly when muscle needs rebuilding upon exercise or attrition [85, 86].

**Figure 4:**
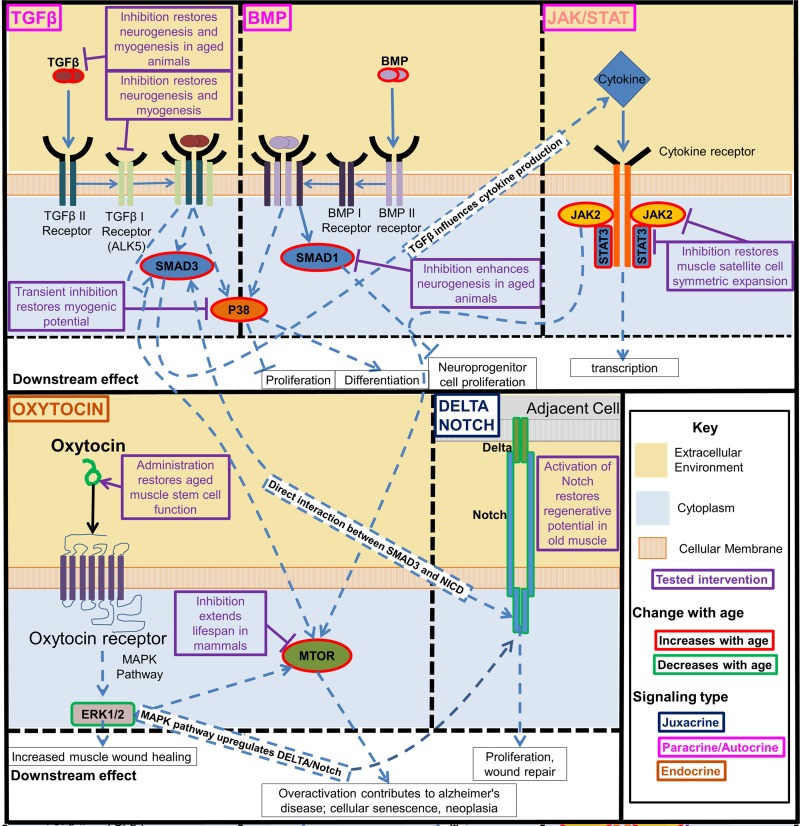
Key regulatory signaling pathways that become deregulated with age The interactive developmentally and evolutionary conserved Notch, TGF-beta/BMP, Jak/Stat, p38, oxytocin/MAPK, and mTOR signaling pathways control function of tissue stem cells and change with age in ways that interfere with tissue maintenance and repair. Experimental modulation of these pathways enhanced the tissue regenerative capacity in old mammals. Beginning clockwise from top left. TGFβ and BMP pathway increase with age and activate pp38, transient inhibition of which is sufficient to restore myogenic potential in muscle stem cells. TGFβ and BMP also separately act through SMADs, inhibiting these SMADs was also found to rescue from some of the negative effects of these pathways pathogenic activation with age. JAK/STAT is a cytokine receptor pathway that increases with age. Many inflammatory cytokines act through this pathway. Inhibition of this pathway restores muscle satellite cell symmetric expansion. Delta/Notch signaling decreases with age, the activation of which in old muscle restores its regenerative potential. Oxytocin signaling decreases with age, restoring this signaling improves aged muscle satellite cell function. These intracellular signaling pathways are highly interactive. TGFβ acts through SMADs to influence downstream cytokine production which act on the Jak/Stat pathway, and SMAD3 and the Notch Intracellular Domain (NICD) interact directly to form a complex in the nucleus that binds to specific DNA sequences [8]. The MAPK/ERK pathway is activated by oxytocin, and the MAPK pathway is known to activate Notch signaling [9]. There is crosstalk between TGFβ signaling, Jak/Stat, and Erk1/2 mediated through mTOR.

Some current stem cell-focused approaches attempt to circumvent the niche aging by normalizing stem cell regulatory signaling to its “youthful intensity” (Figures 3 and 4; as discussed below). The exciting possibility exists that boosting stem cell responses simultaneously in multiple tissues will actually rejuvenate the differentiated cells, such that over time less and less intervention will be needed and the intrinsic age-imposed changes in the differentiated soma would be broadly attenuated or possibly reversed [3]. The approach to boost the regenerative capacity of old tissue stem cells is unrelated to and despite the phenomenon of reduced functionality of induced pluripotent stem cells (iPSCs) that are derived from aged somatic cells (usually fibroblasts) [87], although it would be interesting to examine if differentiated tissue cells derived from rejuvenated old stem cells now have the same functionality as those derived from young tissue.

## Concluding remarks

There is a lot of interest and excitement in the field of aging research and clinical translation is highly anticipated. Even better news is that we do not have to start from scratch and embark on serum or plasma fractionation. Instead, based on published work, agonists and antagonists of specific signaling pathways (some already developed and some FDA approved or in clinical trials), could be used systemically to quickly reset tissue stem cells residing in old organs to their “young” robust regenerative capacity, e.g. overriding the aged niche and its altered molecular cues.

As a proof of principle, this approach has worked for rejuvenation of myogenesis by activation of Notch or MAPK pathways, by attenuation of Jak/Stat signaling, and by neutralization of osteopontin [4, 12, 13, 88, 89].

These and other cell-fate regulatory pathways are highly interactive and some of their interesting and age-responsive cross talks have been revealed [3, 30, 60, 90, 91].

Simultaneous rejuvenation of myogenesis and hippocampal neurogenesis and reduction of inflammation (Beta 2 Microglobulin, B2M levels) in the same old animal have been achieved by one systemically applied TGF-beta receptor inhibitor, and confirmed by genetic approaches [6]. Heterochronic parabiosis also reduced the age-elevated B2M [92]. Down-modulation of BMP signaling enhanced hippocampal neurogenesis in old mice [93], and neutralization of TGF-beta signaling rejuvenated olfactory bulb neurogenesis [94]. A recent study suggests that age-imposed elevation in Smad4 (via deregulation of miR-431) as a mechanism by which both TGF-beta and BMP signaling branches become elevated with age [95].

Similar attenuation of TGF-beta receptor signaling by systemically administered small molecule has greatly improved muscle regeneration at the onset of full diabetes, where muscle stem cell responses are blocked by an upregulation of myostatin [19]. Interestingly, this class of TGF-beta receptor inhibitors is in clinical trials for combating solid tumors (https://clinicaltrials.gov/ct2/results?term=alk5&Search=Search, NCT02160106). TGF-beta signaling activates the p38 pathway, hence Alk5 inhibitors also promise to normalize the elevated, pathological levels of pp38 [96, 97]. Thus multiple positive effects can be expected, particularly in the old.

In addition to the changes in the biochemical cues from local and systemic niches or possibly due to these changes, some stem cells become less sensitive to activating stimuli with age, which might be due to the elevated activity of the molecular target of rapamycin, mTOR pathway [98]. Attenuation of mTOR with rapamycin is being explored as a multi-faceted anti-aging strategy for combatting senescence-associated permanent cell cycle arrest [99, 100] and enhancing tissue regeneration, and rapamycin was suggested to promote stem cell responses [98] and even increase life span in mice [101].

Exploring the secretome of hESCs as a more convenient and translational approach than young blood, a key age-specific role of MAPK has been uncovered; and several novel FGFs have been identified for their pro-regenerative activity on old tissue stem cells in part, by activating the Delta/Notch pathway, and this is conserved between mice and humans [13, 89]. The hESC-secreted pro-regenerative proteins can be isolated via heparin affinity; they enhance regenerative capacity of muscle and neuronal stem cells and are neuroprotective in an *in vitro* model of Alzeimer's disease [13, 89]. A search for the physiologic MAPK agonist that has receptors on muscle stem cells and declines in circulation with aging has identified the FDA approved small peptide oxytocin as a novel anti-aging systemically acting molecule with positive effects on skeletal muscle and bone, mental well being and combating obesity [5, 7, 102–105].

Attenuating inflammatory chemokines and cytokines, including those produced at high levels by senescent cells (SASP), and ablating senescent cells (for example, via the use of their cell-surface markers) are expected to enhance tissue maintenance and regeneration, as well as diminish the risk of the age-related flare of cancers. In this regard, excellent mouse models reporting senescence *in vivo* and enabling selective ablation of p16^high^ cells in live animals have been recently described [106, 107].

These reports demonstrate rational strategies to attenuate and possibly reverse multi-tissue attrition, preventing a number of degenerative and metabolic diseases (sarcopenia, osteoporosis, obesity, diabetes, neuro-degeneration, etc.) as a class, instead of or in addition to approaching each disease individually, Figure 4.
